# College and Hospital Notes

**Published:** 1908-10

**Authors:** 


### COLLEGE AND HOSPITAL NOTES

THE new free dispensary of the German Hospital, Buffalo, was opened September 14, 1908. The following medical and surgical staff has been appointed for the dispensary:

Medicine, L. Bradley Dorr, T. Hubert Wilson, Frank E. Hill; Surgery, J. G. Meidenbauer, James A. MacLeod, H. E. Braner; Gynecology, A. L. Weil, James Porter, Ray H. Johnson; Obstetrics, C. H. W. Auel; Diseases of the eye, William M. Mehl, Gustave A. Hitzel; Diseases of the ear, nose, throat, Francis M. O'Gorman; Diseases of the skin, F. W. Filsinger, Francis E. Fronczak; Orthopedics, L. H. Staples; Nervous diseases, Edward A. Bowermann; Diseases of children, R. C. Mehnert, Joseph Schweitzer, Albert Frey; Clinical pathology, N. K. MacLeod; Genitourinary diseases, William Gardner, Arthur Gibson.

---

FREDERICK COOPER HEWITT who died August 30, 1908, was the wealthiest man in Tioga County. He left an estate estimated to be worth over $5,000,000. His will written by himself, leaves more than $4,000,000 to various institutions, charitable and otherwise. Two million dollars is bequeathed to the New York

Post-Graduate Medical School and Hospital. The will stipulates that should any of his heirs attempt to break the will, he shall forfeit his inheritance, which shall then go to the Metropolitan Museum of Art.

